# Association Between *BRCA* Status and Triple-Negative Breast Cancer: A Meta-Analysis

**DOI:** 10.3389/fphar.2018.00909

**Published:** 2018-08-21

**Authors:** Haixia Chen, Jianming Wu, Zhihong Zhang, Yong Tang, Xiaoxuan Li, Shuangqing Liu, Shousong Cao, Xianzhu Li

**Affiliations:** ^1^Department of Pharmacology, School of Pharmacy, Southwest Medical University, Luzhou, China; ^2^Department of General Medicine, The Affiliated Hospital of Southwest Medical University, Luzhou, China

**Keywords:** Triple-negative breast cancer (TNBC), *BRCA1*, *BRCA2*, mutation, meta-analysis

## Abstract

Triple-negative breast cancer (TNBC) is a subtype of aggressive breast cancer and characterized by a lack of the expression of estrogen receptor, progesterone receptor and human epidermal growth factor receptor 2. *BRCA* genes are tumor-suppressor genes that are involved in DNA damage repair and mutations of *BRCA* genes may increase the risk of developing breast cancer and/or ovarian cancer due to defective DNA repair mechanisms. However, the relationship between *BRCA* status and TNBC needs to be further investigated and validated. The aim of this meta-analysis was to evaluate the association between *BRCA* status and TNBC. We systematically searched the electronic databases of MEDLINE (PubMed), Embase, and Cochrane Library to identify relevant publications from April, 1959 to November, 2017. The data from the studies were examined by a meta-analysis using STATA software to calculate the odds ratio (OR) with 95% confidence interval (CI) by fixed-effect and random-effect models. We identified 16 qualified studies from 527 publications with 46,870 breast cancer patients including 868 *BRCA1* mutations (*BRCA1*^*Mut*^) carriers, 739 *BRCA2* mutations (*BRCA2*^*Mut*^) carriers, and 45,263 non-carriers. The results showed that breast cancer patients with *BRCA1*^*Mut*^ carriers were more likely to have TNBC than those of *BRCA2*^*Mut*^ carriers (OR: 3.292; 95% CI: 2.773–3.909) or non-carriers (OR: 8.889; 95% CI: 6.925–11.410). Furthermore, high expression of nuclear grade and large tumor burden (>2 cm) were significantly more common in breast cancer patients with *BRCA1*^*Mut*^ carriers than those of *BRCA2*^*Mut*^ carriers (OR: 2.663; 95% CI: 1.731–4.097; *P* = 0.211) or non-carriers (OR: 1.577; 95% CI: 1.067–2.331; *P* = 0.157). The data suggest that breast cancer patients with *BRCA1*^*Mut*^ are more likely to have TNBC, high nuclear grade, and larger tumor burden.

## Introduction

Triple-negative breast cancer (TNBC) is an aggressive subtype of breast cancer with a higher risk of both local and distant recurrence and poor overall prognosis and it accounts for about 10–20% of all cases of breast cancer (Foulkes et al., 2010; Ovcaricek et al., 2011; Boyle, 2012). TNBC is characterized by a lack of the expression of estrogen receptor (ER), progesterone receptors (PR) and human epidermal growth factor receptor two (HER2/neu), thus, offers no validated molecular targets for treatment (Onitilo et al., 2009). The *BRCA1* and *BRCA2* genes are tumor-suppressor genes and involved in DNA damage repair and recombination, cell-cycle checkpoint control, apoptosis and transcriptional regulation (Venkitaraman, 2014). Mutations in *BRCA* genes induce defective DNA repair mechanisms, which are associated with the risk of development of breast and/or ovarian cancers (Peng et al., 2016). Some studies showed that *BRCA1* mutation (*BRCA1*^*Mu*^^t^) carriers were more likely to have ER-negative/PR-negative breast cancer (Musolino et al., 2007; Byrski et al., 2008; Kirk, 2010). In contrast, *BRCA2* mutation (*BRCA2*^*Mut*^) carriers seem to share the pathologic characteristics similar to those of patients with normal *BRCA* genes (non-carriers) (Noguchi et al., 1999). However, Comen et al. (2011) found that the association between TNBC and *BRCA* mutations was not only limited to *BRCA1*, but also a significant proportion of women with TNBC had *BRCA2*^*Mut*^. Currently, the relationship between the status of *BRCA* mutation and the statuses of ER, PR, HER2/neu and P53 have been inconsistent (Maegawa and Tang, 2010; Wu et al., 2010). With the development of targeted therapies for breast cancer patients, designation of treatment regimens has become more specific, and breast cancer patients with *BRCA* mutations should be treated differently from the patients without *BRCA* mutations. Therefore, the exact relationship between BRCA status and TNBC needs to be further investigated and validated.

We therefore performed a meta-analysis to investigate the association between the status of *BRCA* mutations and TNBC and the effect of *BRCA*^*Mut*^ on nuclear grade and tumor size in patients with breast cancer.

## Materials and methods

### Data sources and search strategy

We systematically searched the databases of MEDLINE (PubMed, http://www.ncbi.nlm.nih.gov/pubmed/), Embase (http://www.embase.com), and Cochrane Library (www.cochranelibrary.com) for relevant publications of primary studies, and used the following search algorithm: breast cancer, breast carcinoma, mammary cancer, breast tumor and *BRCA1* or *BRCA2, BRCA*, and triple negative breast cancer, TNBC or molecular typing, type or subtype of breast cancer. The databases were searched for the studies published from April, 1959 to November, 2017.

### Study selection

The inclusion criteria were as follows: (a) comparative studies of breast cancer patients with *BRCA1*^*Mut*^, *BRCA2*^*Mut*^, and non-carriers; (b) studies were published as a full paper in English; (c) the statuses of ER, PR and HER2 were measured by immunohistochemistry; and (d) high-quality case-control studies (Newcastle-Ottawa Scale [NOS] score ≥ 7 points). The exclusion criteria were as follows: (a) review articles; (b) study was based on preclinical setting such as cell culture and/or animal models of feline mammary cancer; (c) study did not discuss *BRCA1* and *BRCA2* mutations separately; and (d) study had no inclusion, or duplicated data from other studies.

### Data extraction

Two investigators independently extracted the date from each study including the first author; year of publication; country of study; numbers of subjects with (a) non-carrier with TNBC, (b) non-carriers without TNBC, (c) *BRCA1*^*Mut*^ carrier with TNBC, (d) *BRCA1*^*Mut*^ carrier without TNBC, (e) *BRCA2*^*Mu*^^t^ with TNBC and (f) *BRCA2*^*Mut*^ carrier without TNBC; tumor size and nuclear grade with a standardized form. Additional investigators were consulted when discrepancies were present.

### Population, interventions, comparators, outcomes and study designs (PICOS)

The population from the study is patients with breast cancer. Genetic testing of *BRCA* mutations was performed in these patients. *BRCA* status (*BRCA1* mutations carriers, *BRCA2* mutations carriers, and non-carriers) was compared and the outcomes of incidence of TNBC, expression of nuclear grade and tumor burden (>2 cm) were evaluated in these patients. The study designs were to evaluate the association between *BRCA* status and TNBC as well as the relationship of *BRCA* mutations and the expression of nuclear grade and tumor burden.

### Quality assessment

The quality of each study was independently evaluated by at least two examiners who read each study and scored it according to the NOS criteria (Deeks et al., 2003). The average NOS score was 7.4 points.

### Statistical analysis

The STATA software version 12.0 (Stata Corp, College Station, TX, USA) was used to perform this meta-analysis. Dichotomous outcomes were analyzed using the OR with 95% CI as the summary statistics, as previously described in the Mantel–Haenszel method (Mantel and Haenszel, 1959; Greenland and Robins, 1985). Statistical heterogeneity was evaluated by a *X*^2^ test (Higgins et al., 2003). The Higgins *I*^2^ test measured inconsistency between studies; values of <25, 25–50, and >50% were defined as low, moderate and high, respectively (DerSimonian and Laird, 1986). Data were analyzed with the fixed-effect model for low or moderate consistency and with the random-effect model for high heterogeneity. We also performed sensitivity analysis by omitting specific studies to find potential outliers.

## Results

### Study selection and patient characteristics

**Figure 1:**
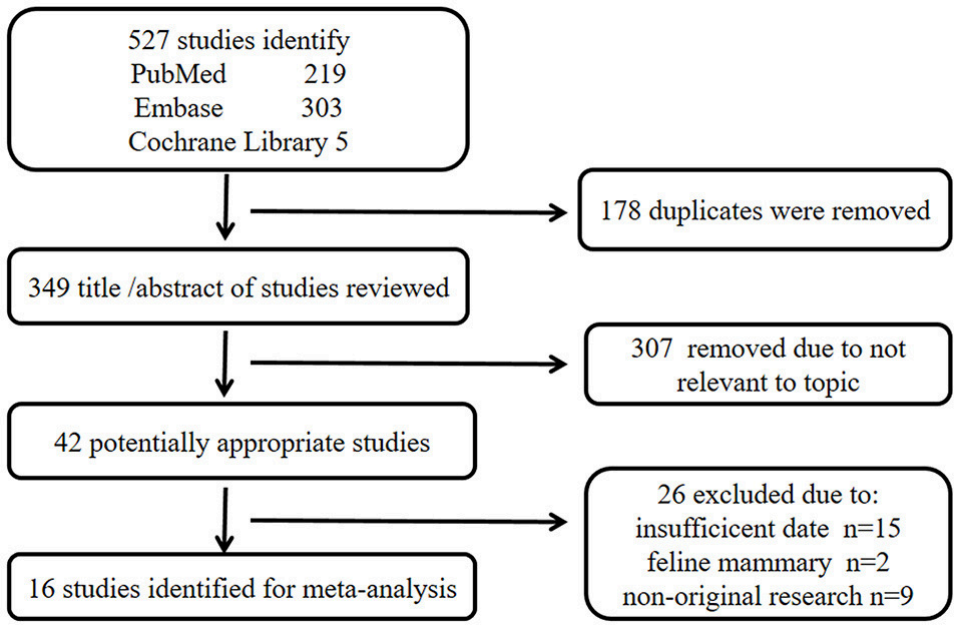
Flow chart for study selection.

A total of 527 publications were identified from the three databases, 219 from PubMed, 303 from Embase, and five from Cochrane Library. The titles and abstracts of all remaining publications (*n* = 349) were reviewed after removing the duplicate publications (*n* = 178) and 307 more publications were excluded as irrelevant to the topic. Next, 26 publications were further excluded for insufficient data (*n* = 15), feline mammary focus (*n* = 2), and non-original research (*n* = 9) after carefully examining the full texts of the remaining 42 publications. Finally, 16 eligible publications were included in the study of meta-analysis (Haffty et al., 2006; Atchley et al., 2008; Kwong et al., 2009; Arun et al., 2011; Comen et al., 2011; Gonzalez-Angulo et al., 2011; Xu et al., 2012; Noh et al., 2013; Li et al., 2014; Yu et al., 2014; Zugazagoitia et al., 2014; Aleskandarany et al., 2015; Gabaldó Barrios et al., 2017; Ghouadni et al., 2017; Ha et al., 2017; Krammer et al., 2017). The screening method and results of the relevant studies are shown in Figure 1 and the main characteristics of participated patients are summarized in Table 1.

**Table 1:** The main characteristics of patients included in the studies.

**First author**	**Country/region**	**Year**	**Median age (year)**	***BRCA1**^***Mut***^**(*****n*****)***		***BRCA2**^***Mut***^**(*****n*****)***		**Non-carrier (*****n*****)**	
				**TNBC**	**Non-TNBC**	**TNBC**	**Non-TNBC**	**TNBC**	**Non-TNBC**
Haffty	USA	2006	NA (NA)	8	2	1	6	13	29
Atchley	USA	2008	43 (21–75)	32	24	7	23	54	337
Kwong	Hong Kong	2009	42 (21–82)	8	4	6	11	45	131
Comen	USA	2011	57.1(NA)	19	6	6	15	39	364
Arun	USA	2011	40 (21–73)	33	19	2	21	NA	NA
Gonzalez-Angulo	USA	2011	51 (27–83)	12	62	3	62	NA	NA
Xu	China	2011	50.6 (29–76)	28	24	8	20	40	232
Noh	Korea	2013	40 (28–52)	16	9	6	16	30	143
Yu	Korea	2014	NA (12–96)	49	31	13	88	6,842	34,758
Zugazagoitia	Spain	2014	32 (NA)	7	5	1	7	NA	NA
Li	China	2014	39.7 (24-64)	18	78	7	78	NA	NA
Aleskandarany	UK	2015	42 (NA)	31	15	2	25	297	1552
Krammer	Germany	2017	44.1(24–82)	128	99	26	185	NA	NA
Ha	Korea	2017	39.7(25–72)	52	47	27	76	NA	NA
Ghouadni	France	2017	52 (38–58)	18	8	3	10	NA	NA
Gabaldó Barrios	Spain	2017	NA	25	13	8	32	43	252

*n, number; NA: not applicable*.

The included studies were conducted in eight countries or regions as USA 5, Korea 3, China 2, Hong Kong 1, UK 1, Germany 1, France 1, and Spain 2, the published date was between 2006 and 2017. 45,870 patients were included in the studies, with the median age ranged from 32.0 to 57.1 years, 868 *BRCA1*^*Mut*^ carriers, 739 *BRCA2*^*Mut*^ carriers, and 45,263 non-carriers (Table 1).

### Association of *BRCA* status and TNBC

**Figure 2:**
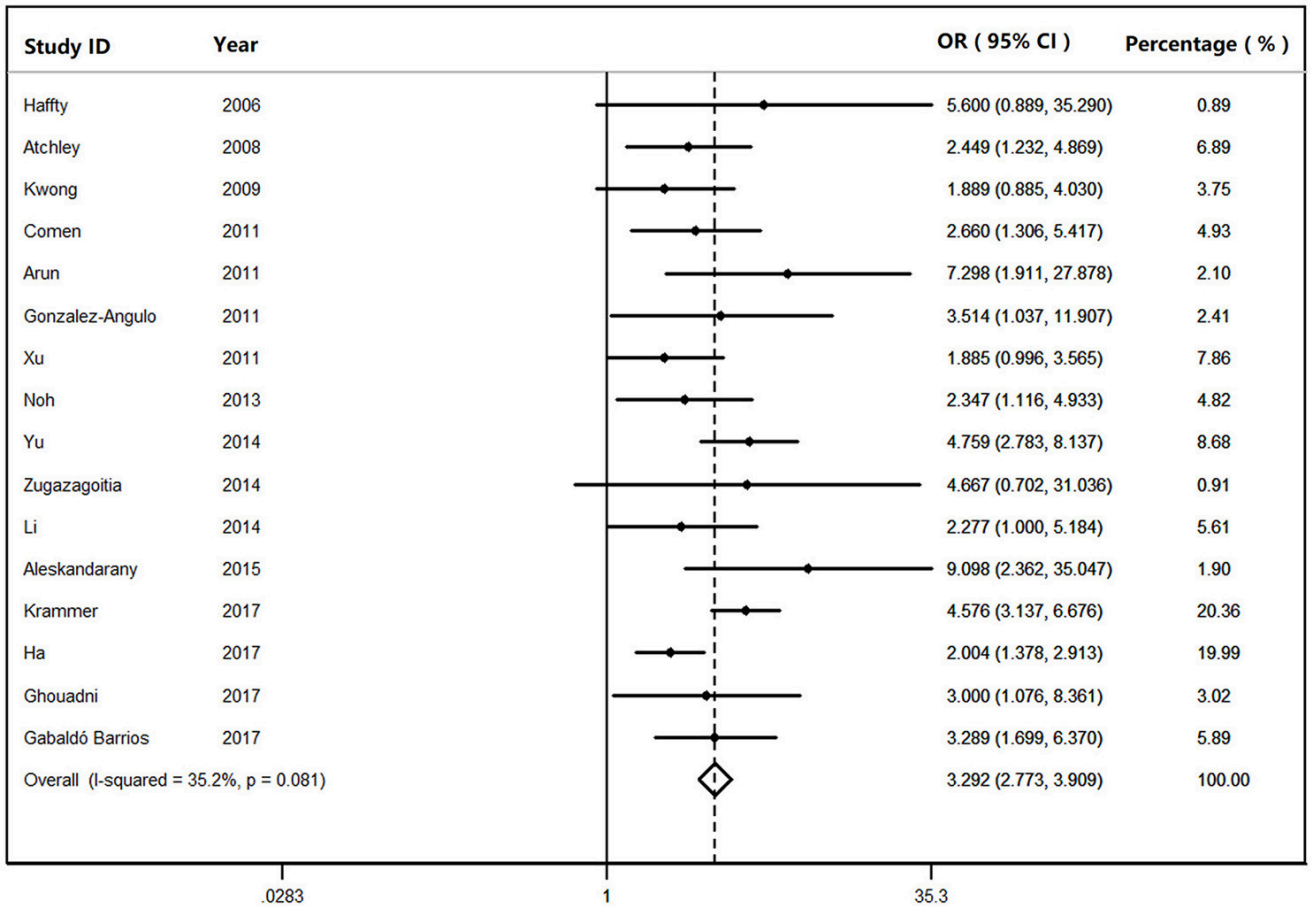
The odds ratio (OR) of *BRCA1* mutations vs. *BRCA2* mutations in patients with TNBC by Forest Plot.

**Figure 3:**
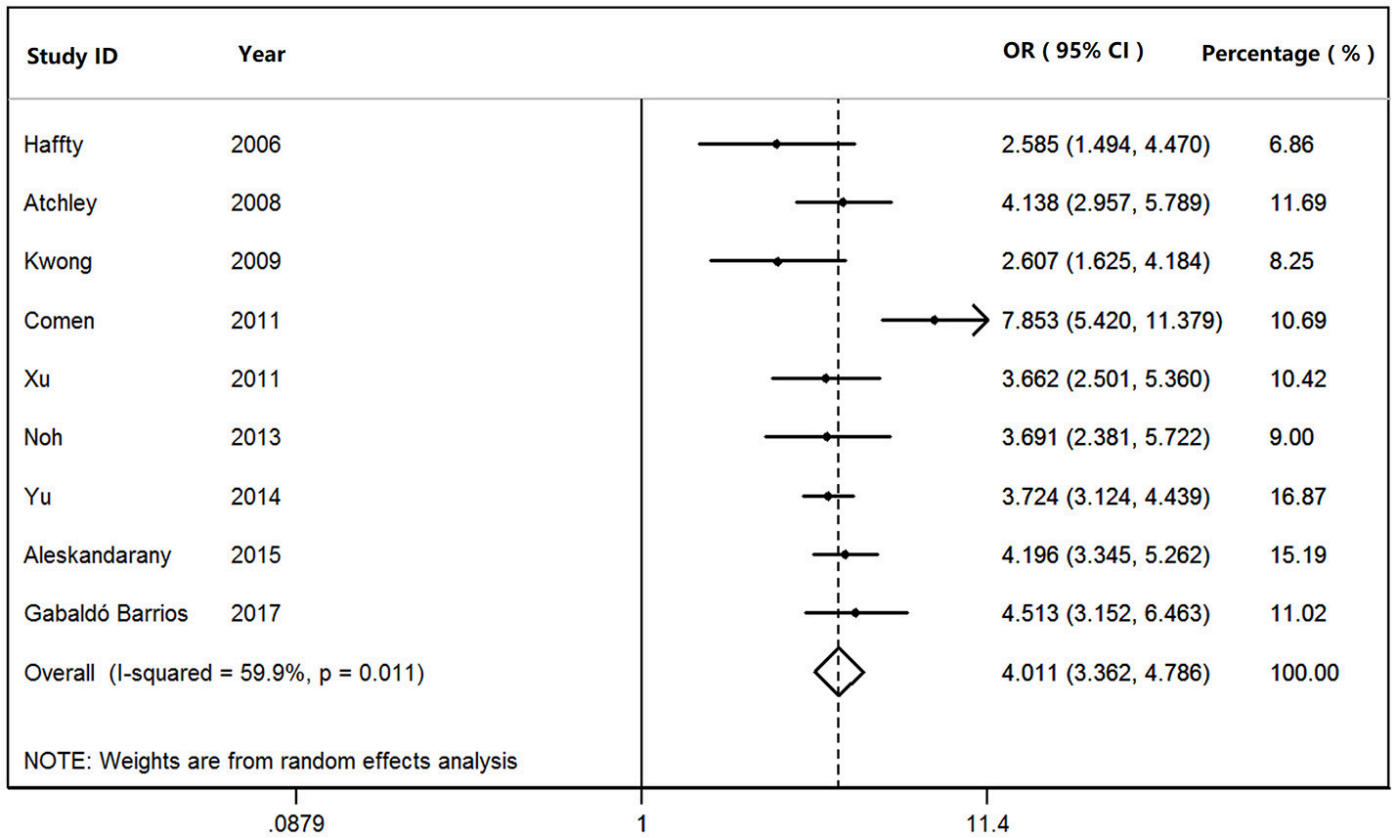
The odds ratio (OR) of *BRCA1* mutations vs. non-carriers in patients with TNBC by Forest Plot.

**Figure 4:**
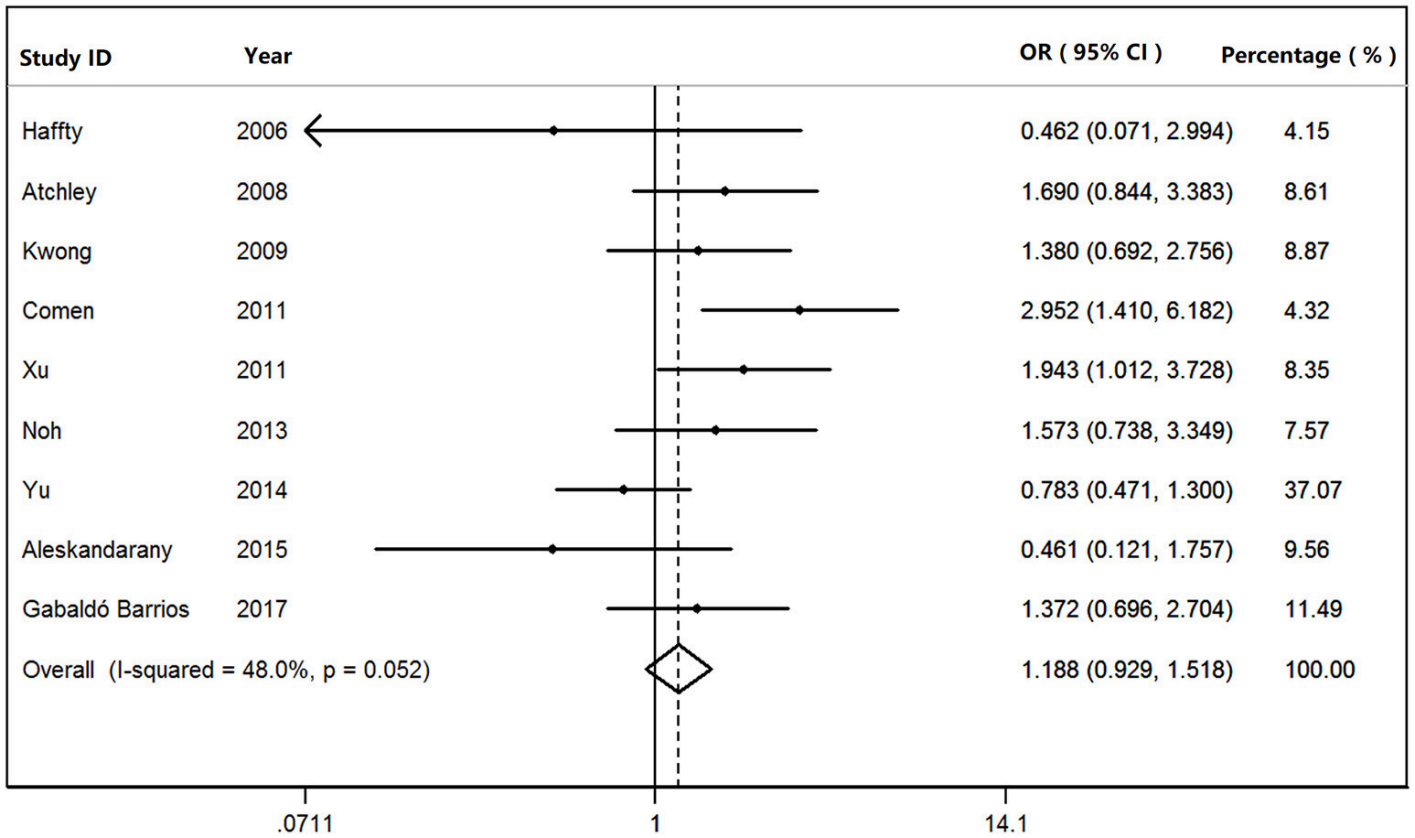
The odds ratio of *BRCA2* mutations vs. non-carriers in patients with TNBC by Forest Plot.

We found that *BRCA1*^*Mut*^ carriers were more likely to have TNBC than those of *BRCA2*^*Mut*^ carriers (OR: 3.292; 95% CI: 2.773–3.909) or non-carriers (OR: 8.889; 95% CI: 6.925–11.410) among the patients with breast cancer (Figure 2). Because heterogeneity was found across the studies (*I*^2^ = 35.2%, heterogeneity *X*^2^ = 23.16; d.f. = 15; *P* = 0.081), the pooled OR was calculated as 3.292 (95% CI: 2.773–3.909) by a fixed-effect model. Furthermore, *BRCA1*^*Mut*^ carriers were significantly more likely to have TNBC than those of non-carriers (Figure 3). There was significant heterogeneity in the studies (*I*^2^ = 59.9%, heterogeneity *X*^2^ = 19.94; d.f. = 8; *P* = 0.011), the pooled OR was calculated as 4.011 (95% CI: 3.362–4.786) by a fixed-effect model. Interestingly, the incidence of TNBC was not significantly different between *BRCA2*^*Mut*^ carries and non-carriers (Figure 4). Because the studies were significantly heterogeneous (*I*^2^ = 48.0%, heterogeneity *X*^2^ = 15.39; d.f. = 8; *P* = 0.052), the pooled OR was calculated as 1.188 (95% CI: 0.929–1.518) by a random-effects model.

### Association of *BRCA* status and nuclear grade or tumor burden

As shown in Table 2, high expression of nuclear grade was more common in the breast cancer patients with *BRCA1*^*Mut*^ carriers than those of patients with *BRCA2*^*Mut*^ carriers (OR: 2.663; 95% CI: 1.731–4.097; *P* = 0.211). Moreover, Tumors were more likely to exceed 2 cm in the breast cancer patients with *BRCA1*^*Mut*^ carriers than those of patients with *BRCA2*^*Mut*^ *carriers* (OR: 1.577; 95% CI: 1.067–2.331; *P* = 0.157).

**Table 2:** Associations between *BRCA* mutation status and tumor size or nuclear grade.

**First author**	***BRCA1**^***Mut***^**(*****n*****)***		***BRCA2**^***Mut***^**(*****n*****)***		***BRCA1**^***Mut***^**(*****n*****)***		***BRCA2**^***Mut***^**(*****n*****)***		**NOS**
	**TS ≤ 2cm**	**TS > 2cm**	**TS ≤ 2cm**	**TS > 2cm**	**NG 1,2**	**NG 3**	**NG 1,2**	**NG 3**	
Haffty	NA	NA	NA	NA	NA	NA	NA	NA	8
Atchley	NA	NA	NA	NA	NA	NA	NA	NA	8
Kwong	6	18	7	2	NA	NA	NA	NA	7
Comen	NA	NA	NA	NA	NA	NA	NA	NA	7
Arun	6	51	5	18	10	45	11	10	8
Gonzalez-Angulo	NA	NA	NA	NA	NA	NA	NA	NA	7
Xu	11	41	8	20	20	32	14	14	7
Noh	15	10	25	7	6	19	20	12	7
Yu	37	38	54	38	20	30	33	29	7
Zugazagoitia	6	18	7	2	NA	NA	NA	NA	7
Li	NA	NA	NA	NA	NA	NA	NA	NA	7
Aleskandarany	24	24	11	16	NA	NA	NA	NA	8
Krammer	NA	NA	NA	NA	65	160	110	105	7
Ha	49	40	41	53	45	54	66	37	7
Ghouadni	NA	NA	NA	NA	NA	NA	NA	NA	7
Gabaldó Barrios	NA	NA	NA	NA	NA	NA	NA	NA	7

*TS, tumor size; NG, nuclear grade; NOS, new castle-ottawa Scale; n, number; NA, not applicable*.

### Sensitivity analyses and publication bias

**Figure 5:**
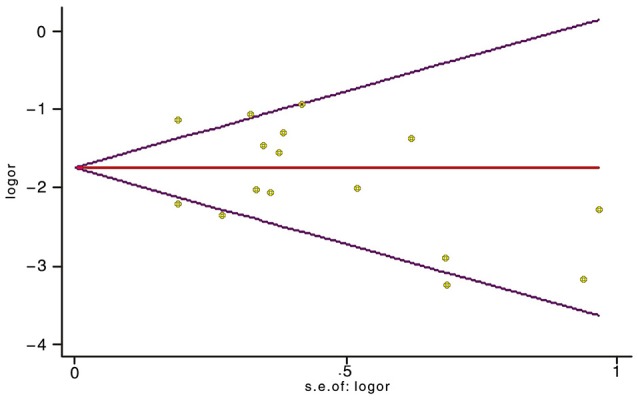
Indication of publication bias for the association between *BRCA1* mutations and *BRCA2* mutations by Begg's Funnel Plot with pseudo 95% confidence limits. The data indicate that there was no obvious indication of publication bias.

**Figure 6:**
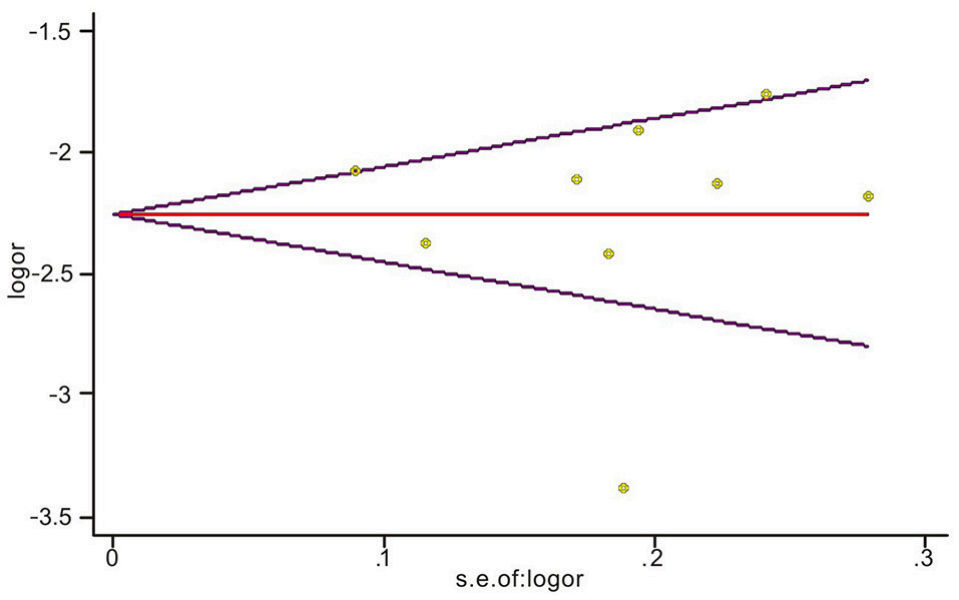
Indication of publication bias for the association between *BRCA1* mutations and non-carriers by Begg's Funnel Plot with pseudo 95% confidence limits. The data indicate that there was no obvious indication of publication bias.

Sensitivity analyses showed that two publications from Li et al. (2014) and Xu et al. (2012) accounted for all the observed heterogeneity. The *I*^2^ was 34.5% when all studies were included in the analysis. However, the *I*^2^ was reduced to 20.5% when the study of Li et al. (2014) was excluded and it was further dropped to 18.1% when the study of Yu et al. (2014) was also removed from the analysis. The results suggest that those two papers significantly influenced the overall analysis. Begg's tests indicated that no publication bias was observed in this meta-analysis for association between *BRCA1*^*Mut*^ and *BRCA2*^*Mut*^ (*P* = 0.499; Figure 5), or between *BRCA1*^*Mut*^ and non-carriers (*P* = 0.348; Figure 6).

## Discussion

We investigated the association between *BRCA* status and TNBC (a subtype of breast cancer) and the characteristics of breast cancer patients with *BRCA1*^*Mut*^ and *BRCA2*^*Mut*^ using a meta-analysis. The currently specific criteria of guidelines from the National Comprehensive Cancer Network (NCCN) for test of *BRCA1*^*Mut*^ and *BRCA2*^*Mut*^ include patients' ages at diagnosis and their family members; family histories of breast, ovarian, pancreatic and prostate cancers, and diagnosed TNBC (National Comprehensive Cancer Network, 2017). Up to date, approximately 300 mutations within the *BRCA1*^*Mut*^ gene have been identified, including small insertions, deletions and non-sense mutations, most of them lead to functionally inactive proteins (Miki et al., 1994; Simard et al., 1994). *BRCA2* is a tumor suppressor gene that mediates the repair of chromosomal damage (Yoshida and Miki, 2004). In the present study of meta-analysis, we found that TNBC was more common among the breast cancer patients with *BRCA1*^*Mut*^ than those of patients with *BRCA2*^*Mut*^ (OR: 3.292; 95% CI: 2.773–3.909) or non-carriers (OR: 8.889; 95% CI: 6.925–11.410). In an unselected cohort study in 77 patients with TNBC, it was found that 15 (19.5%) had *BRCA* mutations including 12 (15.6%) in *BRCA1* (one somatic) and 3 (3.9%) in *BRCA2* (Gonzalez-Angulo et al., 2011). In addition, a significantly lower risk of relapse was found in TNBC patients with *BRCA* mutations (Gonzalez-Angulo et al., 2011).

The underlying mechanism that links *BRCA1*^*Mut*^ to ER negativity has been the focus of ongoing investigations. Hosey et al. (2007) discovered that *BRCA1*^*Mut*^ tumors fail to express ER due to the loss of *BRCA1*-mediated transcriptional activation of estrogen receptor 1 (ESR1). Reduction or absence of *BRCA1* in breast cancer occurs through several mechanisms including hypermethylation of the *BRCA1* promoter, loss of heterozygosity, and transcriptional regulation of *BRCA1* (Catteau et al., 1999; Baldassarre et al., 2003). However, the exact mechanism for the transcription of *BRCA1* is highly complex and remains unknown. Further studies are needed to gain insight into the interaction between *BRCA1* and ER, and its potential effects on the expressions of PR and HER2.

*BRCA2*^*Mut*^ breast cancer has the pathologic features similar to those of sporadic breast cancers (Lee et al., 2010). The incidence of TNBC was not significantly different between patients with *BRCA2*^*Mut*^ and non-carriers (OR: 1.203; 95% CI: 0.871–1.660). Hosey et al. (2007) suggested that breast cancer patients with *BRCA2*^*Mut*^ were unlikely to be ER-deficient because of the ability of estrogen metabolites to induce loss of the second *BRCA1* allele, thus, estrogen may somehow facilitate the survival of *BRCA1*-deficient cells in hormonally responsive tissues.

Interestingly, in the present study, we found that a high nuclear grade was also more common in the tumors from *BRCA1*^*Mut*^ patients than in those of patients with *BRCA2*^*Mut*^ carriers (OR 2.663; 95% CI: 1.731–4.097; *P* = 0.211) and the tumors with *BRCA1*^*Mut*^ were more likely to have nuclear grade three than those of tumors with *BRCA2*^*Mut*^. This finding is consistent with the earlier studies in the literature (Musolino et al., 2007; Li et al., 2008; Xu et al., 2012). We also found that the tumors with *BRCA1*^*Mut*^ were more likely to exceed 2 cm than those of tumors with *BRCA2*^*Mut*^ ($OR 1.577; 95% CI: 1.067–2.331), although several studies have reported that the sizes of tumors were not significantly different between the tumors with *BRCA1*^*Mut*^ and the tumors with *BRCA2*^*Mut*^ (Xu et al., 2012; Noh et al., 2013; Yu et al., 2014). The observed difference of tumor size may be due to different clinical and pathological characteristics from the tumors with *BRCA1*^*Mut*^ and *BRCA2*^*Mut*^ leading to different prognosis in the patients with *BRCA* mutations. The findings may be significant with valuable information for oncologists to better understand the role of *BRCA* mutations in breast cancer patients and optimal treatment of TNBC.

Some limitations of the study should be acknowledged in this meta-analysis. The methods of assessing ER/PR-negative status were varied among the studies. Most studies defined ER/PR-negative specimens as having <10% immunoreactive cells, whereas newer immunohistochemistry guidelines have used a threshold of <1%. *BRCA* mutation tests also lack uniformity, which may affect the outcomes. Therefore, selection bias was inevitable.

## Conclusion

The present study suggests that TNBC was more common among the breast cancer patients with *BRCA1*^*Mut*^ tumors than those of patients with *BRCA2*^*Mut*^ tumors or non-carriers. Furthermore, a high expression of nuclear grade and large tumor burden (> 2cm) were significantly more common in *BRCA1*^*Mut*^ patients than that of *BRCA2*^*Mut*^ patients. The study provides valuable information for clinicians to better understand the role of *BRCA* mutations in breast cancer patients for providing optimal treatment and improving outcome clinically.

## Author contributions

HC, JW, SC, and XZL designed the study and analyzed the data; HC, JW, ZZ, and XZL collected and assembled the data; HC, JW, YT, XXL, SL, SC, and XZL reviewed, analyzed, and interpreted the data. HC, JW, SC, and XZL assessed the risk of bias; HC, JW, and XZL wrote the first draft and SC wrote the final version of the manuscript. All authors discussed the results and contributed to the manuscript.

### Conflict of interest statement

The authors declare that the research was conducted in the absence of any commercial or financial relationships that could be construed as a potential conflict of interest.
